# Supplementary material for: Hyperpiliation, not loss of pilus retraction, reduces Pseudomonas aeruginosa pathogenicity
Source: Microbiol Spectr. 2025 Feb 25;13(4):e02558-24. doi: 10.1128/spectrum.02558-24 (PMC11960060; doi:10.1128/spectrum.02558-24)
Supplement: Supplemental figures — Fig. S1 to S10. [file spectrum.02558-24-s0001.docx]

**SUPPLEMENTARY DATA FOR:**

**Hyperpiliation, not loss of pilus retraction, reduces *Pseudomonas aeruginosa* pathogenicity**

Sara L.N. Kilmury, Katherine J. Graham, Ryan P. Lamers, Lesley T. MacNeil, and Lori L. Burrows


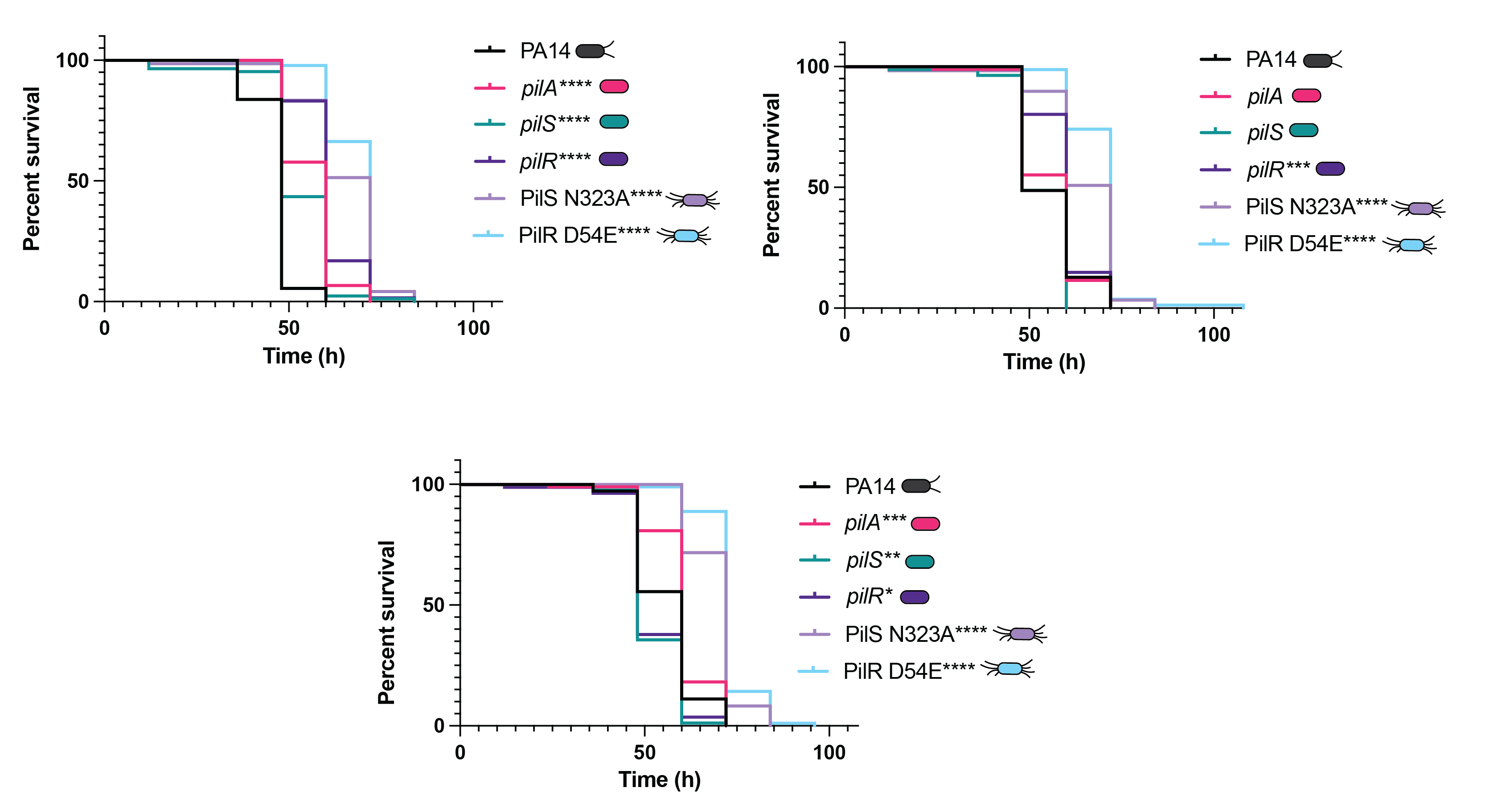
Department of Biochemistry and Biomedical Sciences and the Michael G. DeGroote Institute for Infectious Disease Research, McMaster University, Hamilton, ON Canada

**Supplementary Figure S1. Hyperpiliated mutants of PA14 have reduced pathogenicity towards *C. elegans.*** Hyperpiliated PilS N323A and PilR D54E point mutants of the highly virulent *P. aeruginosa* PA14 strain are less pathogenic than wild type or its isogenic non-piliated *pilA*, *pilS*, or *pilR* mutants. These data show that loss of pathogenicity in those backgrounds is not strain specific. Asterisks (* p<0.05, ** p<0.01, *** p<0.001, **** p<0.0001) indicate strains that were significantly different from PA14 by Gehan-Breslow-Wilcoxon test. Three biological replicates are shown.


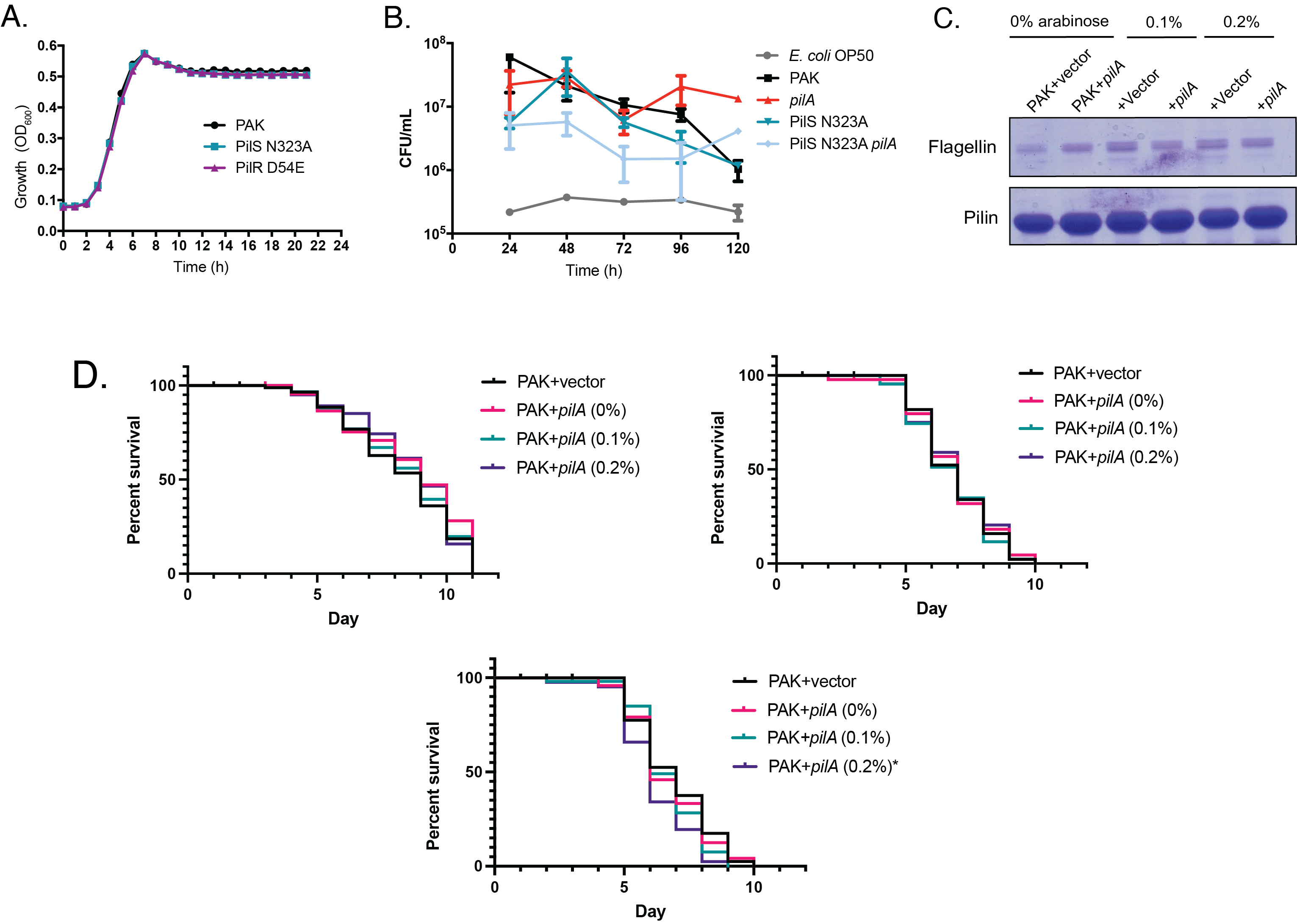


**Supplementary Figure S2. The growth and *C. elegans* colonization capacity of hyperpiliated PAK point mutants in slow killing medium is similar to wild type and overexpression of PilA *in trans* in PAK does not cause hyperpiliation or reduce pathogenicity.**

**A.** To verify that reduced pathogenicity was not the result of a growth defect, the wild type and mutants were grown in liquid slow killing medium for 24 h at 37^o^C. There was no difference between strains in growth rate or terminal optical density at 600 nm (OD_600_). The mean and standard error of three independent biological replicates (nine total samples) are shown. **B.** Bacterial survival in the gut of *C. elegans* was measured over the course of 5 days for WT PAK, *pilA*, PilS N323A and PilS N323A *pilA*. This graph is a representative dataset from triplicate experiments. No significant differences in persistence of the four strains were apparent. **C.** PilA was overexpressed *in trans* from an inducible vector in WT PAK at the indicated concentrations of the inducer, arabinose. Sheared surface protein preparations showed that surface piliation was unaltered at any of the arabinose concentrations tested. This gel is a representative dataset from triplicate experiments. **D.** Arabinose-induced overexpression of PilA *in trans* did not alter pathogenicity of PAK in the *C. elegans* slow killing model. Asterisks (* p<0.05) indicate strains that were significantly different from PAK by Gehan-Breslow-Wilcoxon test. Three biological replicates are shown.


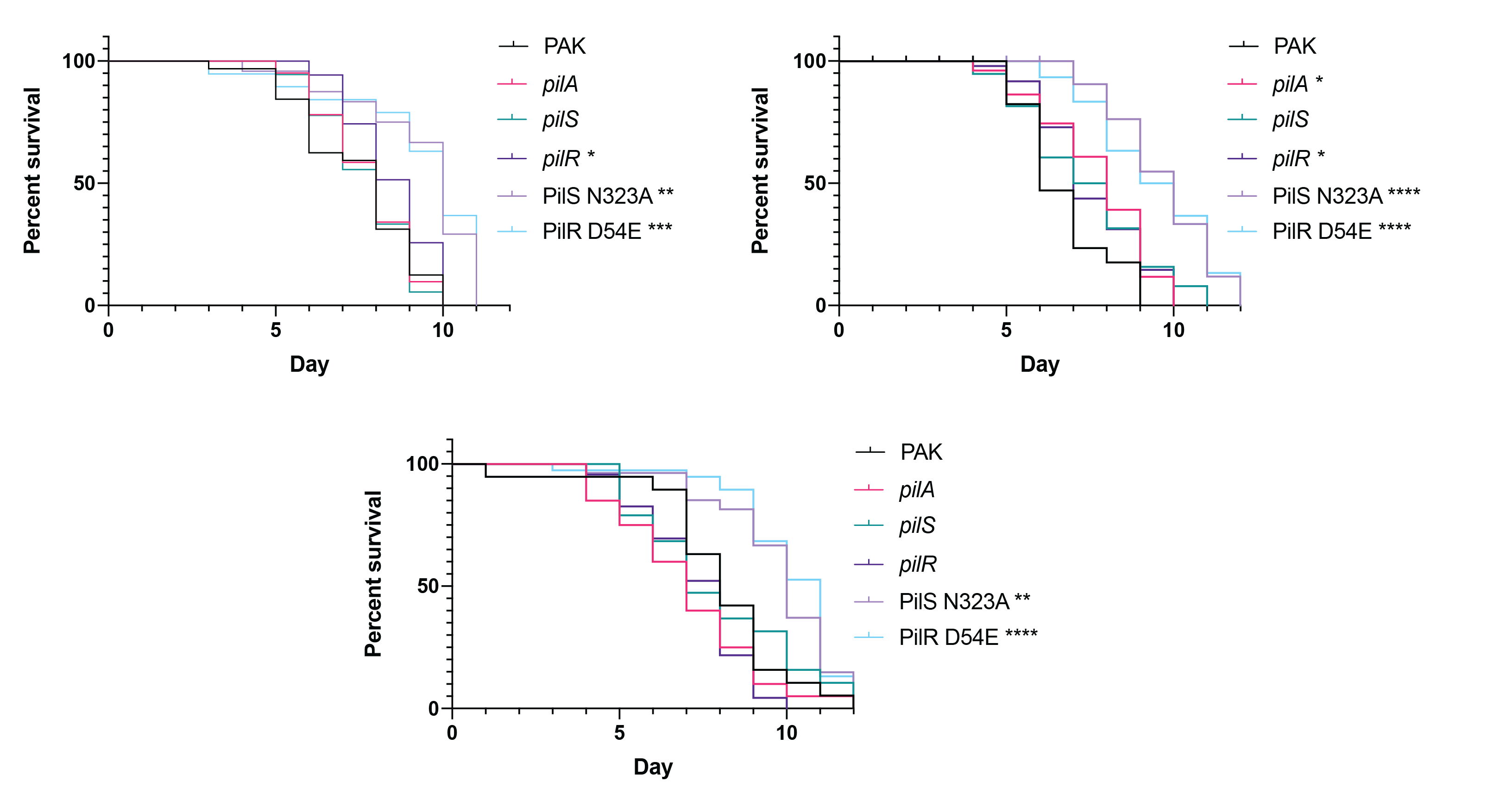


**Supplementary Figure S3. Three biological replicates for Fig 2A.**

Slow killing assays for PAK *pilA*, *pilS*, *pilR*, PilS N323A, and PilR D54E mutants. The hyperpiliated PilS N323A and PilR D54E mutants are significantly less pathogenic in slow killing assays than wild type PAK or its isogenic *pilA*, *pilS*, or *pilR* mutants; all of which lack surface pili. Asterisks (* p<0.05, *** p<0.001, **** p<0.0001) indicate strains that were significantly different from PAK by Gehan-Breslow-Wilcoxon test.


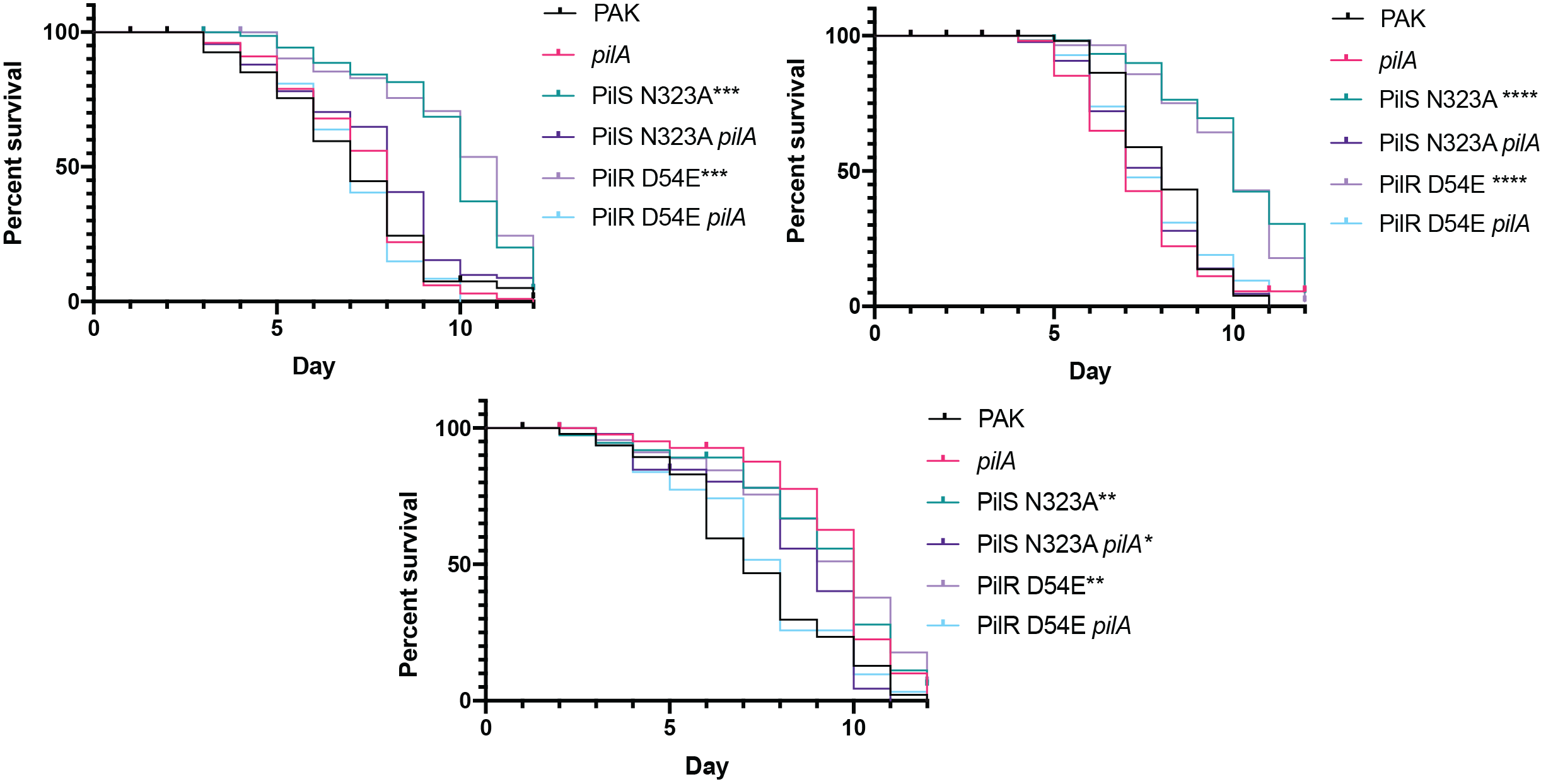


**Supplementary Figure S4. Three biological replicates for Fig 2B.**

Slow killing assays for PAK *pilA*, PilS N323A, PilS N323A *pilA*, PilR D54E, and PilR D54E *pilA* mutants. Deletion of *pilA* in the PilS N323A or PilR D54E backgrounds restores pathogenicity to levels similar to PAK and the *pilA* control. Asterisks (* p<0.05, *** p<0.001, **** p<0.0001) indicate strains that were significantly different from PAK by Gehan-Breslow-Wilcoxon test.


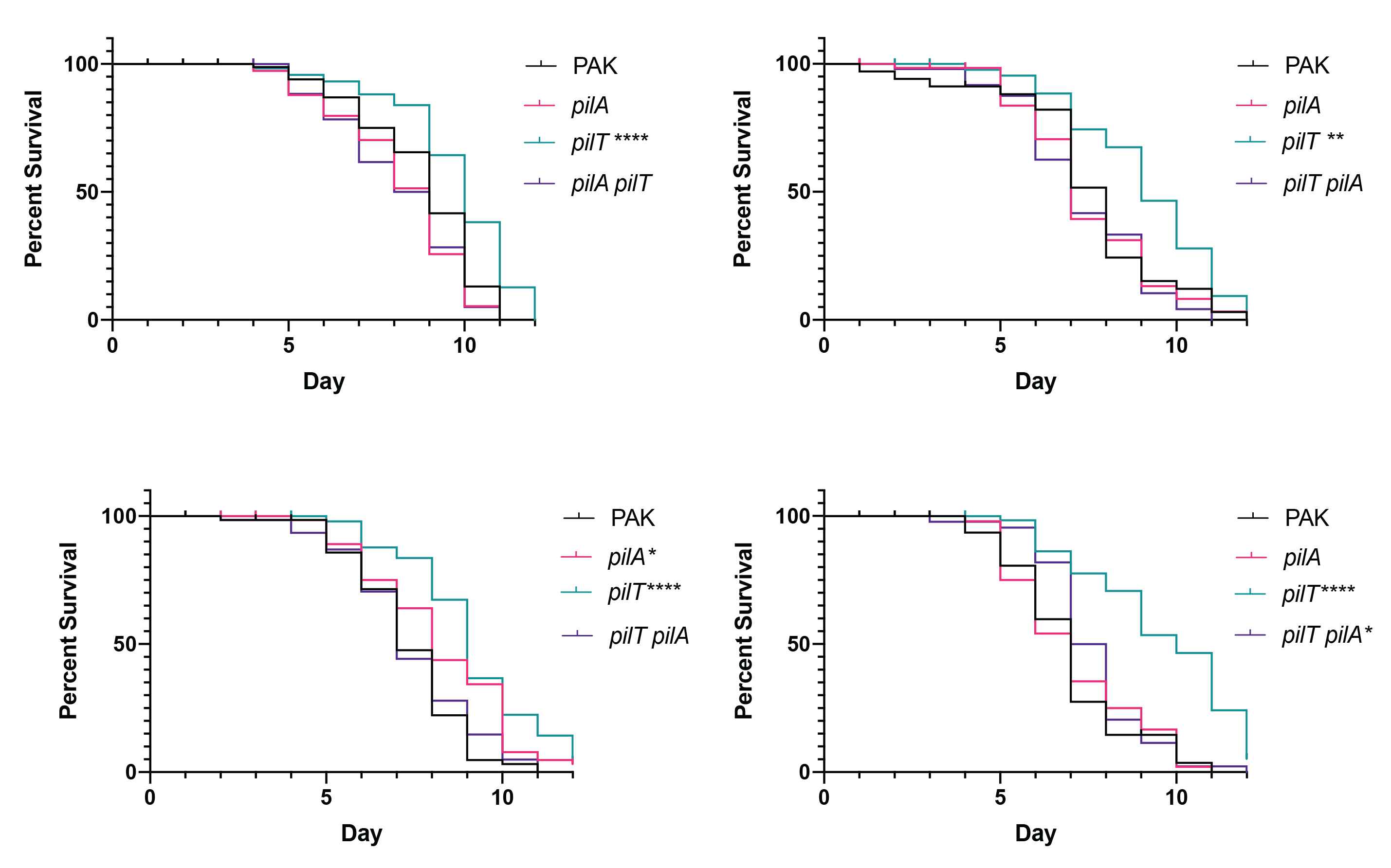


**Supplementary Figure S5. Four biological replicates for Fig 2C.**

Slow killing assays for PAK *pilA*, *pilT*, and *pilA pilT* mutants. Loss of *pilA* in the *pilT* background, which is significantly less pathogenic than wild type, restores pathogenicity to levels similar to wild type and the *pilA* control. Asterisks (* p<0.05, *** p<0.001, **** p<0.0001) indicate strains that were significantly different from PAK by Gehan-Breslow-Wilcoxon test.


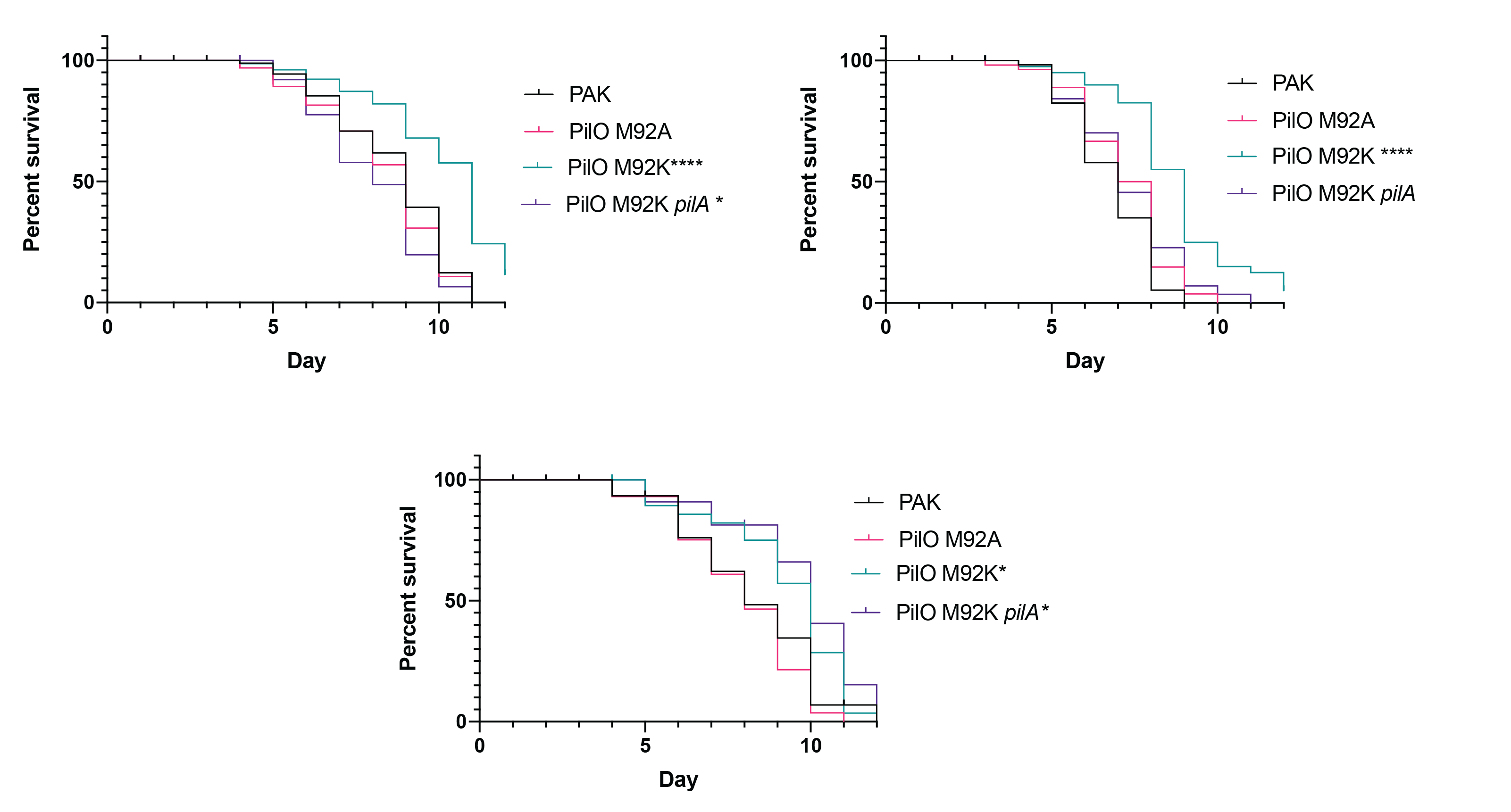


**Supplementary Figure S6. Three biological replicates for Fig 2D.**

Slow killing assays for PAK PilO M92A, PilO M92K, and PilO M92K *pilA* mutants. The hyperpiliated PilO M92K mutant is less pathogenic than PAK or an isogenic PilO M92A mutant, and pathogenicity is restored by deletion of *pilA* in the M92K background. Asterisks (* p<0.05, *** p<0.001, **** p<0.0001) indicate strains that were significantly different from PAK by Gehan-Breslow-Wilcoxon test.


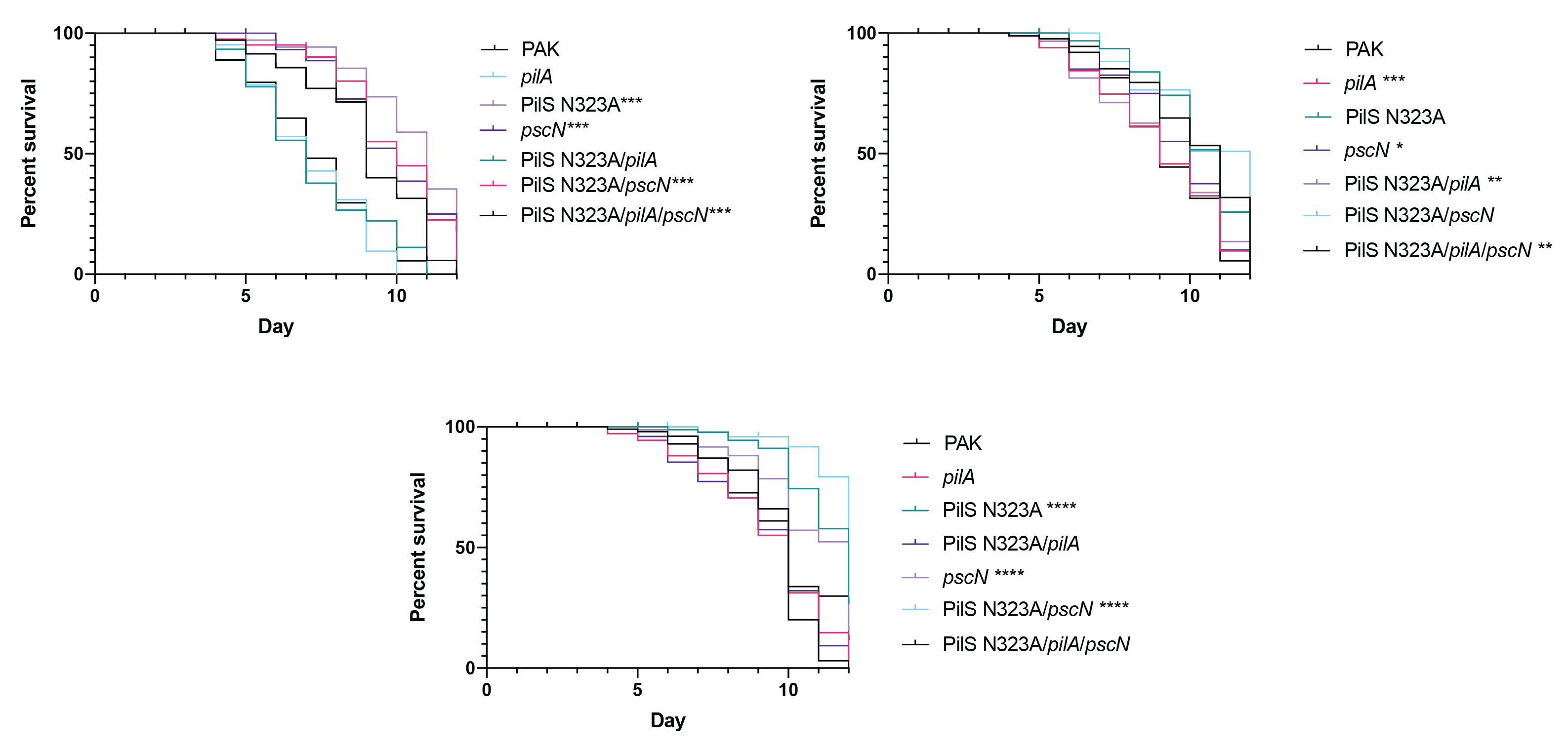


**Supplementary Figure S7. Three biological replicates for Fig 4A.**

Slow killing assays for PAK *pilA*, PilS N323A, *pscN*, PilS N323A *pilA*, PilS N323A *pscN*, and PilS N323A *pilA pscN* mutants. Deletion of *pscN*, encoding the T3SS ATPase, reduces pathogenicity of PAK towards *C. elegans*, showing that virulence of this strain is T3SS-dependent. Combining the *pscN* and PilS N323A mutations does not further decrease virulence. While loss of *pilA* in the N323A background increases pathogenicity, further deletion of *pscN* in the N323A *pilA* background reduces pathogenicity, confirming that virulence is T3SS-dependent. Asterisks (* p<0.05, *** p<0.001, **** p<0.0001) indicate strains that were significantly different from PAK by Gehan-Breslow-Wilcoxon test.


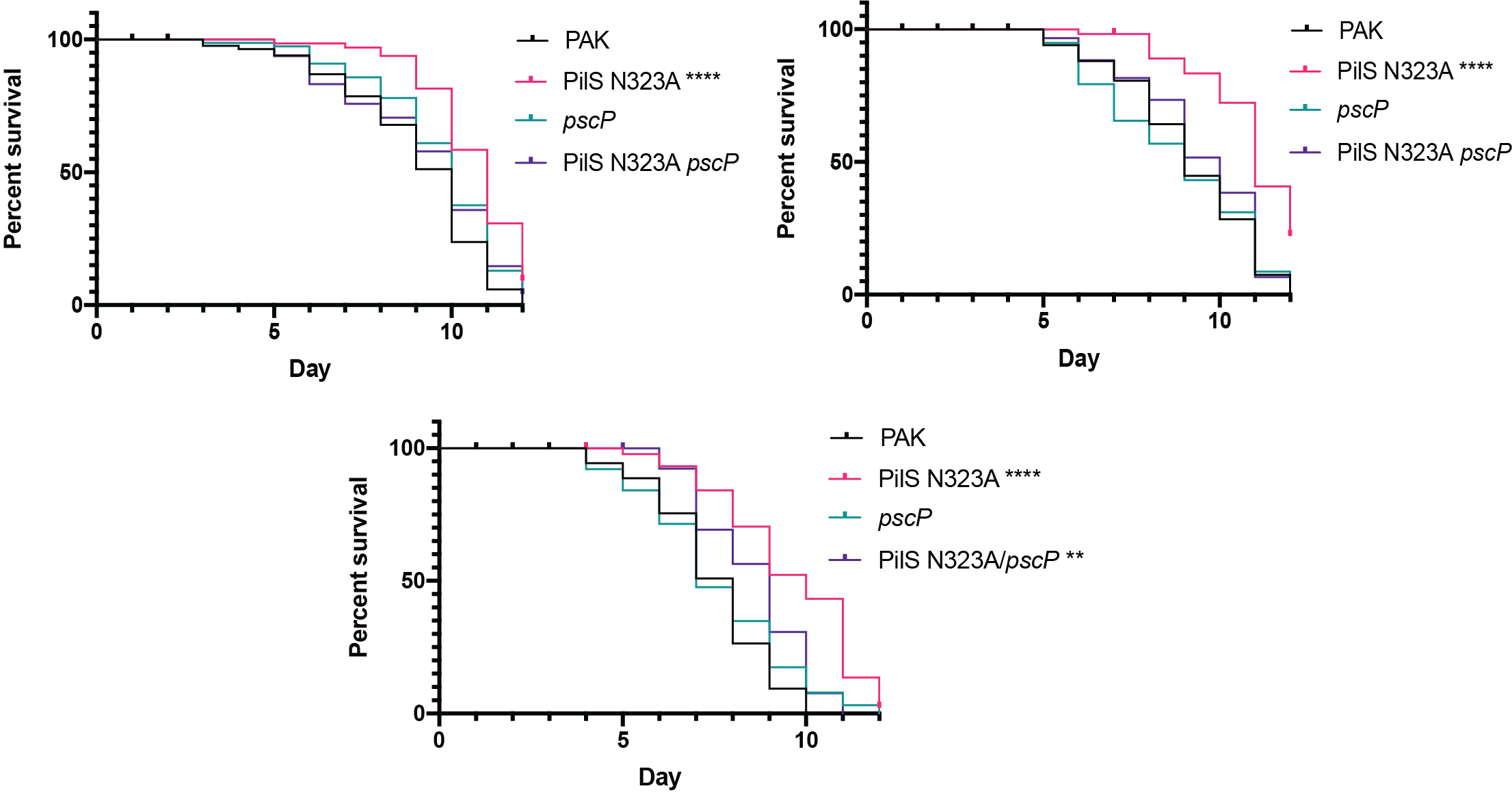


**Supplementary Figure S8. Three biological replicates for Fig 4B.**

Slow killing assays for PAK PilS N323A, *pscP*, and PilS N323A *pscP* mutants. While deletion of *pscP*, encoding the T3SS ruler protein that controls needle length, does not impair virulence of PAK towards *C. elegans*, deletion of this gene in the hyperpiliated N323A background increases pathogenicity. Asterisks (* p<0.05, *** p<0.001, **** p<0.0001) indicate strains that were significantly different from PAK by Gehan-Breslow-Wilcoxon test.

**
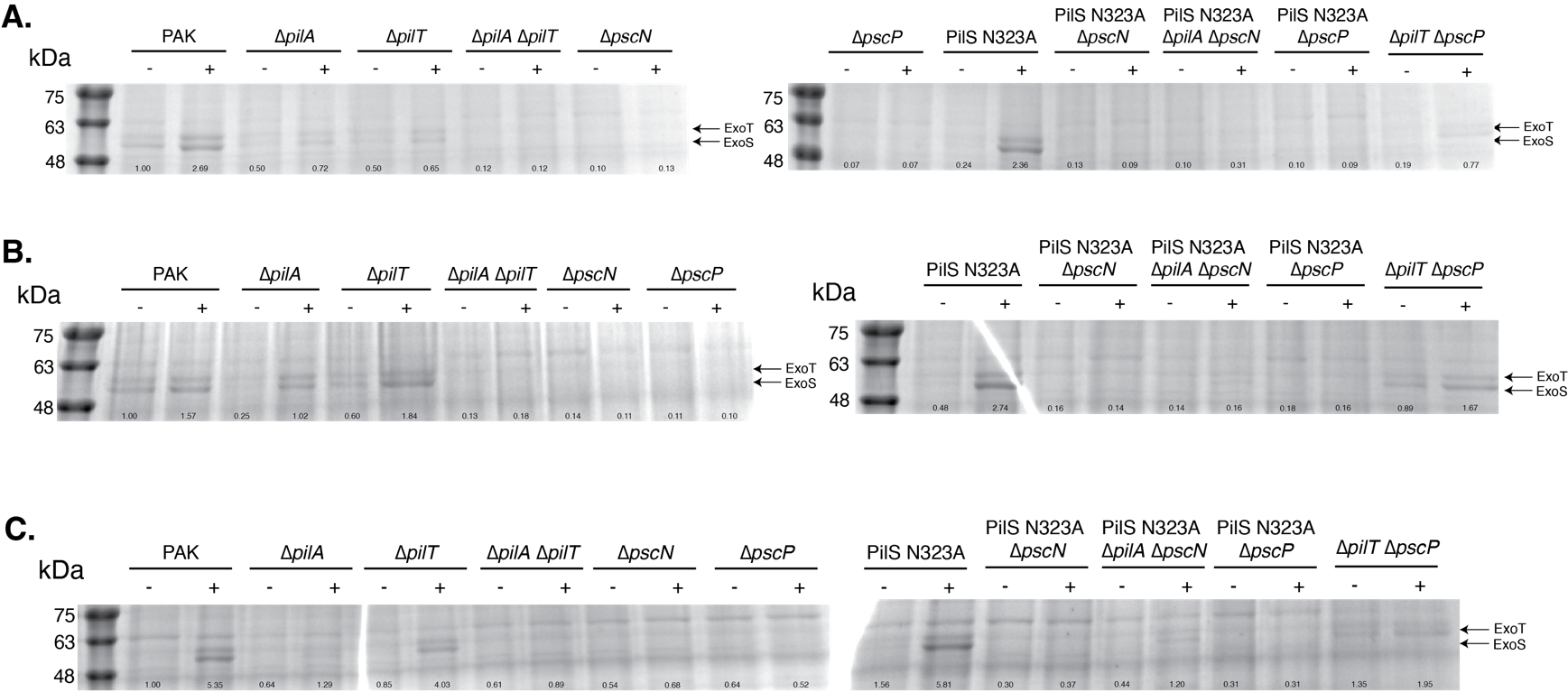
**

**Supplementary Figure S9: Exotoxin secretion assay for T3S/hyperpiliated mutants used in this study.**

Strains were grown in T3S-inducing conditions and supplemented with either pBADGr (-) or pBADGr-*exsA* (+). ∆*pilT* and PilS N323A strains had comparable secretion of ExoS/T to WT, while ∆*pilA*, ∆*pilA ∆pilT*, and ∆*pilT* ∆*pscP* strains had less detectable ExoS/T in culture supernatants. Excluding the ∆*pilT* ∆*pscP* strain, strains harbouring a deletion in PscN or PscP had no detectable ExoS/ExoT in culture supernatants. Three biological replicates are shown. Pixel density of ExoS/T relative to WT (-) are displayed in each lane.


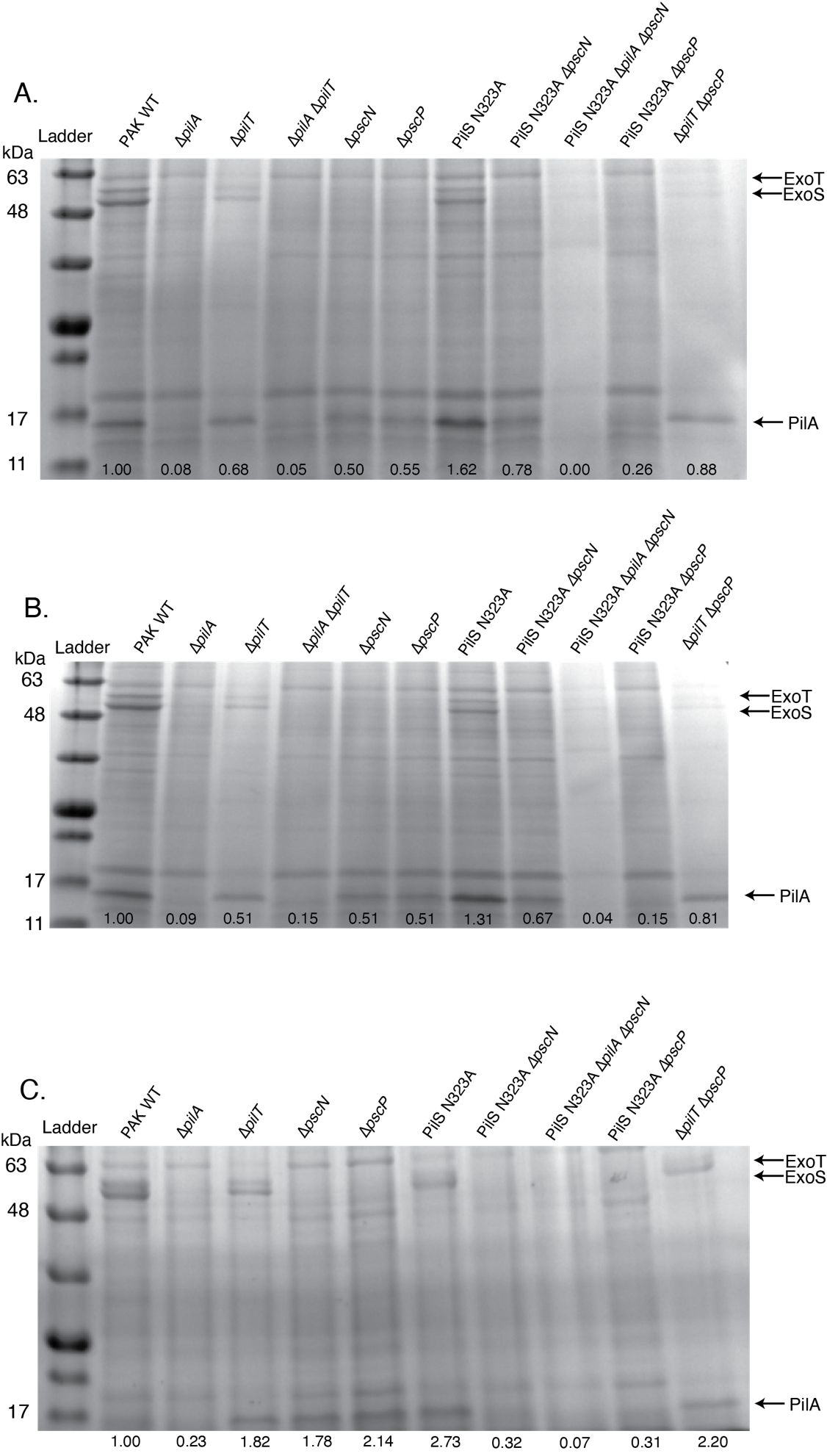


**Supplementary Figure S10: Pilin expression in supernatants of the T3S/hyperpiliated mutants used in this study.**

Strains were grown in T3SS-inducing conditions and supplemented with pBADGr-*exsA*. While WT, *pilT*, *pscN*, *pscP*, PilS N323A, PilS N323A *pscN*, and *pilT pscP* strains had detectable PilA (15.5 kDa), PilA was undetected in strains harbouring a genetic lesion in *pilA* and the PilS N323A *pscP* mutant. Pixel density of PilA relative to WT are displayed in each lane.
