# Supplementary material for: Hyperpiliation, not loss of pilus retraction, reduces Pseudomonas aeruginosa pathogenicity
Source: Microbiol Spectr. 2025 Feb 25;13(4):e02558-24. doi: 10.1128/spectrum.02558-24 (PMC11960060; doi:10.1128/spectrum.02558-24)
Supplement: Supplemental tables — Tables S1 and S2. [file spectrum.02558-24-s0002.docx]

**Table S1: Strains and plasmids used in this study**

| Strain | Characteristics | Source |
| --- | --- | --- |
| *E. coli* strains | | |
| *E. coli* DH5α | F- φ80*lac*ZΔM15 Δ(*lac*ZYA-*arg*F)U169 *rec*A1 *end*A1 *hsd*R17(rk-, mk+) *pho*A *sup*E44 *thi*-1 *gyr*A96 *rel*A1 λ | Invitrogen |
| *E. coli* SM10 | *thi-1 thr leu tonA lacY supE recA*::RP4-2- Tc::Mu (KmR) | (72) |
| *E. coli* OP50 |  | (L.T. MacNeil) |
| *P. aeruginosa* strains | | |
| PAK WT | WT, Group II T4AP | (J. Boyd) |
| PAK WT + pBADGr-*exsA* | WT with pBADGr containing *exsA* | (This work) |
| PAK WT + pBADGr | WT with pBADGr | (This work) |
| PA14 WT | WT, Group III T4AP | (G.A. O'Toole) |
| PAK *pilA* | Chromosomal deletion of *pilA* | (This work) |
| PAK *pilA* + pBADGr-*exsA* | Chromosomal deletion of *pilA* with pBADGr containing *exsA* | (This work) |
| PAK *pilA* + pBADGr | Chromosomal deletion of *pilA* with pBADGr | (This work) |
| PAK *pilS* | Chromosomal deletion of *pilS* | (This work) |
| PAK *pilR* | Chromosomal deletion of *pilR* | (This work) |
| PAK *pilT* | Chromosomal deletion of *pilT* | (This work) |
| PAK *pilT* + pBADGr-*exsA* | Chromosomal deletion of *pilT* with pBADGr containing *exsA* | (This work) |
| PAK *pilT* + pBADGr | Chromosomal deletion of *pilT* with pBADGr | (This work) |
| PAK PilS N323A | Chromosomal substitution of PilS phosphatase motif residue N323 to alanine | (11) |
| PAK PilS N323A + pBADGr-*exsA* | Chromosomal substitution of PilS phosphatase motif residue N323 to alanine with pBADGr containing *exsA* | (This work) |
| PAK PilS N323A + pBADGr | Chromosomal substitution of PilS phosphatase motif residue N323 to alanine | (This work) |
| PAK PilR D54E | Chromosomal substitution of PilR phosphorylation site D54 to glutamic acid | (This work) |
| PAK PilR D54E + pBADGr-*exsA* | PAK with a chromosomal substitution of PilR phosphorylation site D54 to glutamic acid with pBADGr containing *exsA* | (This work) |
| PAK PilR D54E + pBADGr | Chromosomal substitution of PilR phosphorylation site D54 to glutamic acid with pBADGr | (This work) |
| PAK PilO M92A | Chromosomal substitution of residue M92 to alanine in PilO | (41) |
| PAK PilO M92K | Chromosomal substitution of residue M92 to lysine in PilO | (41) |
| PAK PilO M92K/*pilA* | PilO M92K strain with chromosomal *pilA* deletion | (This work) |
| PAK *pilA*::FRT/*pilT*::FRT | FRT scar in *pilA* and FRT scar at position 540 in *pilT* | (11) |
| PAK *pilA*::FRT/*pilT*::FRT + pBADGr-*exsA* | FRT scar in *pilA* and FRT scar at position 540 in *pilT* with pBADGr containing *exsA* | (This work) |
| PAK *pilA*::FRT/*pilT*::FRT + pBADGr | FRT scar in *pilA* and FRT scar at position 540 in *pilT* with pBADGr | (This work) |
| PAK PilS N323A/*pilA* | Disrupted PilS phosphatase motif and clean *pilA* deletion | (This work) |
| PilR D54E/*pilA* | Altered PilR phosphorylation site and *pilA* deletion | (This work) |
| PAK *pscN* | Chromosomal deletion of the Type III secretion ATPase *pscN* | (This work) |
| PAK *pscN* + pBADGr-*exsA* | Chromosomal deletion of the Type III secretion ATPase *pscN* with pBADGr containing *exsA* | (This work) |
| PAK *pscN* + pBADGr | Chromosomal deletion of the Type III secretion ATPase *pscN* with pBADGr | (This work) |
| PAK *pscN*/PilS N323A | Chromosomal deletion of Type III secretion ATPase and chromosomal substitution of PilS residue N323 to alanine | (This work) |
| PAK *pscN*/PilS N323A + pBADGr-*exsA* | Chromosomal deletion of Type III secretion ATPase and chromosomal substitution of PilS residue N323 to alanine with pBADGr containing *exsA* | (This work) |
| PAK *pscN*/PilS N323A + pBADGr | Chromosomal deletion of Type III secretion ATPase and chromosomal substitution of PilS residue N323 to alanine with pBADGr | (This work) |
| PAK *pscN*/PilS N323A/*pilA* | Chromosomal deletion of *pscN* and *pilA* combined with PilS N323A substitution | (This work) |
| PAK *pscN*/PilS N323A/*pilA*+ pBADGr-*exsA* | Chromosomal deletion of *pscN* and *pilA* combined with PilS N323A substitution and pBADGr containing *exsA* | (This work) |
| PAK *pscN*/PilS N323A/*pilA* + pBADGr | Chromosomal deletion of *pscN* and *pilA* combined with PilS N323A substitution and pBADGr | (This work) |
| PAK *pscP* | Chromosomal deletion of *pscP* | (This work) |
| PAK *pscP* + pBADGr-*exsA* | Chromosomal deletion of *pscP* with pBADGr containing *exsA* | (This work) |
| PAK *pscP* + pBADGr | Chromosomal deletion of *pscP* with pBADGr | (This work) |
| PAK *pscP*/PilS N323A | Chromosomal deletion of *pscP* combined with PilS N323A substitution | (This work) |
| PAK *pscP*/PilS N323A + pBADGr-*exsA* | Chromosomal deletion of *pscP* combined with PilS N323A substitution and pBADGr containing *exsA* | (This work) |
| PAK *pscP*/PilS N323A | Chromosomal deletion of *pscP* combined with PilS N323A substitution and pBADGr | (This work) |
| PAK *pilT/pscP* | Chromosomal deletion of *pilT* and *pscP* | (This work) |
| PAK *pilT/pscP* + pBADGr-*exsA* | Chromosomal deletion of pilT and pscP with pBADGr containing exsA | (This work) |
| PAK *pilT/pscP* + pBADGr | Chromosomal deletion of pilT and pscP with pBADGr | (This work) |
| PA14 *pilA* | Chromosomal deletion of *pilA* | (This work) |
| PA14 *pilS* | Chromosomal deletion of *pilS* | (This work) |
| PA14 *pilR* | Chromosomal deletion of *pilR* | (This work) |
| PA14 PilS N323A | Chromosomal substitution of PilS residue N323 to alanine | (This work) |
| PA14 PilR D54E | Chromosomal substitution of PilR residue D54 to glutamic acid | (This work) |
| PAO1 *fliC pilA* + pBADGr-*exsA* | Chromosomal deletion of *fliC* and *pilA* with pBADGr containing PAK *exsA* | (This work) |
| PAO1 *fliC pilA* + pBADGr | Chromosomal deletion of *fliC* and *pilA* with pBADGr | (This work) |
| *C. elegans* species | | |
| *C. elegans* N2 | WT Bristol strain | (L.T. MacNeil) |
|  |  |  |
| Vector | **Characteristics** | **Source** |
| pEX18Gm | Suicide vector used for gene replacement | (11) |
| pBADGr | Broad host range arabinose inducible vector used for complementation; ori *araC-PBAD* Gmʳ *mob*⁺ | (6) |
| pBADGr+PAK *pilA* | pBADGr expressing PAK PilA | (73) |

**Table S2: Primers used in this study**

| **Primer Name** | **Sequence (5'→3')^a^** |
| --- | --- |
| *pilA* F1 | GCG GAA TTC GTG TTG GCG GAC CAG CTT |
| *pilA* R1 | GCA CCC GGG GCC TTT TTG AGC TTT CAT |
| *pilA* F2 | GCA CCC GGG CCG AAA GGT TGC TCT AAG TAA |
| *pilA* R2 | ATT GCA TGC ATT GCC GAG GCC CGG |
| *pilS* F1 | GTT GAA TTC GCC GGA AAA CCA GGA TC |
| *pilS* R1 | GTT GGA TCC CAG ACG GAG GAT GCG TTG |
| *pilS* F2 | GTT GGA TCC GGA AGG CGG CGG CTG C |
| *pilS* R2 | GTT AAG CTT ACT GAT GTA GAC CGG CGC |
| *pilR* F1 | GTC AGA ATT CCT CCC GTC GCC GCC AGG C |
| *pilR* R1 | TGA CGG ATC CGA CGA TCA GGG CTT TTT G |
| *pilR* F2 | GTC AGG ATC CCG CCT GAA AAA GCT GGG C |
| *pilR* R2 | TGA CAA GCT TGG CCT GGA ACT GCC CGT G |
| *pilT* F1 | CTT AGA ATT CGA TGA ACG CTA TGC G |
| *pilT* R1 | CTT AGG ATC CGT TCA TGA TGT CGT AG |
| *pilT* F2 | CTT AGT CGA CCA CGA GAT CAT GAT C |
| *pilT* R2 | CTT AAA GCT TCA GGG TGT TCT TCA G |
| PilR D54E F | GAC CTG TGC CTC ACC GAG ATG CGC CTG CCG GAC |
| PilR D54E R | GTC CGG CAG GCG CAT CTC GGT GAG GCA CAG GTC |
| *pscN* F1 | ATT GAA TTC GGT GGG CGA TCA GCG CCT |
| *pscN* R1 | ATT GGA TCC CGA TGG CGT GGC GCA TCC |
| *pscN* F2 | ATT GGA TCC AGC GAT TAC GCA CAG GCC |
| *pscN* R2 | GGC AAG CTT TTC CAG TTC GCC TTC CTC |
| *pscP* F1 | GTC AGA GCT CCG CAC AGG CCT GCG CGC A |
| *pscP* R1 | GCT AGG ATC CGT CGG ACG ACA CGA GCG |
| *pscP* F2 | GCT AGG ATC CCG CTC GCG GCA ACG TCG C |
| *pscP* R2 | GCT AAA GCT TCA GGC CGG GCC ATT GCA G |
| *exsA* F | ATT AGA ATT CGT TCT TAT AAT ATG CAA GGA GCC |
| *exsA* R | ATT AAA GCT TTC AGT TAT TTT TAG CCC GGC ATT C |

**^a^ – Restriction sites are underlined.**
